# Supplementary material for: An autonomous drone swarm for detecting and tracking anomalies among dense vegetation
Source: Commun Eng. 2025 Nov 27;4:205. doi: 10.1038/s44172-025-00546-8 (PMC12660990; doi:10.1038/s44172-025-00546-8)
Supplement: Supplementary file 2 — Supplementary Information [file 44172_2025_546_MOESM2_ESM.pdf]

An Autonomous Drone Swarm for Detecting and Tracking Anomalies among Dense Vegetation

Rakesh John Amala Arokia Nathan[1], Sigrid Strand[2], Daniel Mehrwald[1], Dmitriy Shutin[2], Oliver Bimber[1]*

[1]Department of Computer Science, Johannes Kepler University Linz, 4040 Linz, Austria.
[2]Institute of Communications and Navigation Communications Systems, German Aerospace Center, 82234 Oberpfaffenhofen-Wessling, Germany.

*Corresponding Author: oliver.bimber@jku.at, Johannes Kepler University Linz.

SUPPLEMENTARY MATERIALS

Supplementary Note 1: Computing Integral Images with Airborne Optical Sectioning

Unlike conventional methods that rely on constructing 3D point clouds or meshes through complex computations, AOS leverages image-based rendering for 3D visualization [1]. This approach circumvents challenges such as inaccurate correspondence matches and prolonged processing times commonly encountered in photogrammetry. At its core, AOS operates by sampling the optical signal, using wide synthetic apertures that typically range from 30 to 100 meters in diameter. This is achieved through unstructured video images captured by camera drones, facilitating optical sectioning via image integration. The wide aperture signal results in a shallow depth of field, which leads to significant blurring of out-of-focus occluders, such as leaves, branches, and foliage. In contrast, objects in focus remain sharply registered.

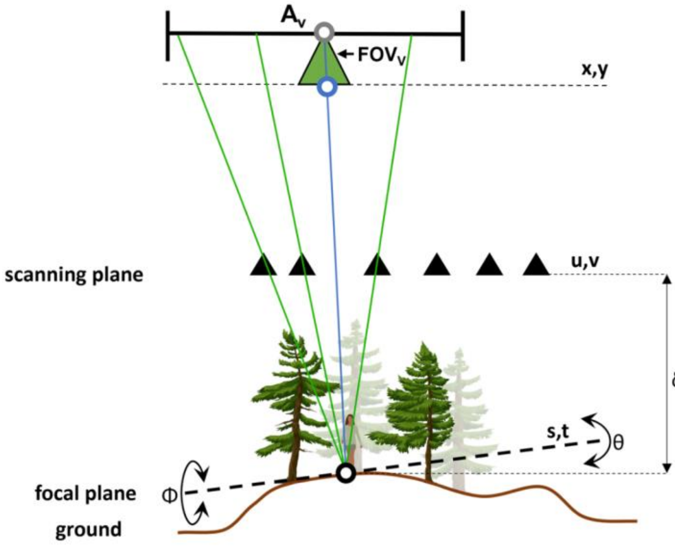

Algorithm S1 Integral Image Computation

Require 1: focal plane at orientation θ, Φ and distance δ
Require 2: virtual camera with field of view FOV_v and in synthetic aperture A_v

```
for all points on focal plane at s,t
    intgeral_{s,t} = 0
    for all drones on scanning plane at u,v
        if ray_{s,t,u,v} within FOV_v then
            intgeral_{s,t} = intgeral_{s,t} + ray_{s,t,u,v}
        end if
    end for
end for
```

Figure S1. Visualization Technique of AOS Using Image-Based Rendering [2]. Note that $ray_{s,t,u,v}$ in the pseudocode provides the intersected pixel of the drone image captured at position u,v.

Figure S1 illustrates the general computational process of an AOS integral image, including pseudocode [3] calculated for a pre-defined virtual camera by means of unstructured light-field rendering [1]. The virtual camera, represented by the green triangle, is predefined with its parameters (position, orientation, focal plane, synthetic aperture, and field of view) within the shared three-dimensional coordinate system the drone defines (black triangles with *u,v* coordinates in the scanning plane). Integral images are computed via ray summation for each point *s,t* in the focal plane (black circle) across the synthetic aperture A_v that are within the field of view FOV_v. The focal plane is at a distance δ and has an orientation θ, Φ. The value of each ray is determined by projecting *s,t* into the corresponding drone's perspective. Repeating this for all points (*s,t*) in the focal plane and projecting them into the perspective of the virtual camera (blue circle) results in the integral image. From a light-field rendering perspective [2], this corresponds to a 4D two-plane ray parameterization (*u,v,s,t*). If A_v covers the entire scanning plane (*u,v*), the integral images create an extremely shallow depth of field, which results in blurring not only of occluding objects, such as trees, but also of any points on the ground that are not aligned with the synthetic focal plane. In this case, the position of the virtual camera must be in the center of the scanning plane to avoid clipping at the borders. The distance of the virtual camera from the scanning plane, as well as its field of view, scales the integral image. In our case, this distance is 0, and FOV_v is identical to the field of view of the drones' cameras.

Supplementary Note 2: Anomaly Detection

Color anomaly detection methods play a crucial role in analyzing multispectral images by identifying pixel regions that exhibit a low probability of occurrence within the background landscape, thereby classifying them as outliers. These techniques are pivotal in a wide range of remote-sensing applications, for instance, in agriculture, wildlife observation, surveillance, and search and rescue operations, to name only a few.

In aerial image analysis, computational methods such as anomaly detection and classification are crucial for automating target detection tasks. Due to their robustness and independence of large amounts of training data, model-based (statistical) anomaly detection approaches, exemplified by the Reed–Xiaoli (RX) detector [4,5], offer distinct advantages over classification methods. Unsupervised color anomaly detection methods, including the extensively used RX detector, have been widely adopted [4,5,6,7,8,9], making RX detection a classical and dependable technique for identifying anomalies in multispectral images [4,5]. Our previous work [10] has investigated and evaluated several variants of the RX detector and further enhanced its applicability and performance in various remote-sensing applications.

Notably, the RX anomaly detector is effective for both hyperspectral and multispectral images. It characterizes the background of an image by using a covariance matrix, and calculates the RX score based on the Mahalanobis distance between a pixel under test and the background. The RX score is defined as:

$$\alpha_{RX}(r) = (r - \delta)^T K^{-1} (r - \delta), \tag{1}$$

where r represents the spectral vector of the pixel under evaluation, δ denotes the spectral mean vector of the background, and K signifies the covariance matrix.

For all raw images sampled by each drone during one iteration, we determine binary anomaly masks by applying the RX anomaly detector. Specifically, for each image, an RX score is computed for every pixel based on the background statistics as described in Eqn 1. A threshold is then applied to generate a binary mask that highlights anomalous regions. Here, the top *t%* of all image pixels with the highest RX scores α_{RX} are identified as anomalies, where *t* is referred to as the RX threshold. Integrating these detected anomalies using AOS enhances occlusion removal and suppresses outliers, thereby increasing the likelihood of detecting occluded targets. In our experiments, we used *t*=99.75%.

Supplementary Note 3: Results

Table S1 and Figure S2 summarize quantitative measures and results of Experiment I, which was carried out on 2024-04-28 at 10:25 a.m.

Table S1. Quantitative measures and results of Experiment I. GPS coordinates of ground-truth target positions and the swarm's estimates for each PSO iteration. The detections with confidence scores of a simplified pre-trained classifier (YOLO-World v2 [11], restricted to only two classes: persons and vehicles). The ground distances between the swarm's target estimates and the target's ground-truth bounding box (in the case of the vehicle: 5.1 m x 1.7 m). Note that a distance of 0 m indicates an estimate inside the target's bounding box. Note that a dash ('-') for the YOLO confidence score indicates a "no-target-detection" event and therefore no confidence score is provided for the corresponding entries. The detections with precision and recall of our approach and of the legacy method. Note also that 'no' in the detection column indicates a false negative, while 'wrong' indicates a false positive.

iteration	ground-truth target coordinates (center)		estimated target position coordinates		YOLO-World confidence score	distance between ground-truth target (bounding box) and estimated target position (m)	detection (our approach)	detection (legacy method)
	latitude	longitude	latitude	longitude				
1	47.98941973	11.30628726	47.98941821	11.30628845	0.32	0.00	correct	correct
2	47.98942995	11.30631276	47.98942350	11.30631761	0.42	0.00	correct	correct
3	47.98943632	11.30632874	47.98943858	11.30634106	0.39	0.00	correct	correct
4	47.98944557	11.30635300	47.98944529	11.30636605	0.37	0.00	correct	correct
5	47.98945612	11.30637875	47.98946051	11.30639534	0.41	0.00	correct	correct

6	47.98946689	11.30640281	47.98946676	11.30642200	0.41	0.00	correct	correct
7	47.98948250	11.30643450	47.98948778	11.30645084	0.45	0.00	correct	correct
8	47.98950306	11.30647428	47.98951061	11.30649118	0.51	0.03	correct	correct
9	47.98952258	11.30651218	47.98952344	11.30654532	0.35	0.00	correct	correct
10	47.98954222	11.30655072	47.98954638	11.30657044	0.29	0.00	correct	correct
11	47.98956698	11.30659993	47.98957185	11.30663059	0.41	0.00	correct	correct
12	47.98958653	11.30663867	47.98959293	11.30665646	0.39	0.00	correct	correct
13	47.98960563	11.30667559	47.98960871	11.30669489	0.24	0.00	correct	correct
14	47.98962773	11.30671741	47.98963617	11.30674233	0.32	0.14	correct	correct
15	47.98964822	11.30675547	47.98965066	11.30677327	0.18	0.00	correct	correct
16	47.98967796	11.30681005	47.98969452	11.30685567	0.35	1.35	correct	correct
17	47.98971144	11.30686939	47.98971714	11.30689425	0.05	0.00	correct	correct
18	47.98975440	11.30694106	47.98975558	11.30695593	0.02	0.00	correct	wrong
19	47.98978826	11.30699791	47.98978357	11.30650340	-	-	wrong	wrong
20	47.98981371	11.30704023	47.98969410	11.30675132	-	-	wrong	wrong
21	47.98982113	11.30705255	47.98970493	11.30677071	-	-	wrong	wrong
22	47.98982973	11.30706627	47.98982854	11.30706187	0.02	0.00	correct	correct
23	47.98982953	11.30706651	47.98982797	11.30706996	0.11	0.00	correct	correct

24	47.98982948	11.30706637	47.98983076	11.30707458	0.25	0.00	correct	correct
25	47.98982100	11.30705328	47.98982333	11.30706339	0.27	0.00	correct	correct
26	47.98982744	11.30706254	47.98983317	11.30707618	0.26	0.00	correct	correct
27	47.98985017	11.30709386	47.98985975	11.30711146	0.2	0.25	correct	correct
28	47.98986313	11.30711069	47.98987267	11.30712451	0.32	0.24	correct	correct
29	47.98989026	11.30713525	47.98990702	11.30714054	0.22	0.37	correct	correct
30	47.98992275	11.30713663	47.98993755	11.30713157	0.49	0.11	correct	correct
31	47.98994756	11.30711424	47.98996236	11.30709688	0.56	0.49	correct	correct
32	47.98997274	11.30707392	47.98997732	11.30706476	0.57	0.00	correct	correct
33	47.98998409	11.30705607	47.99000177	11.30702909	0.65	1.22	correct	correct
34	47.99001827	11.30701216	47.99003127	11.30698436	0.42	1.27	correct	correct
35	47.99004377	11.30698143	47.99005056	11.30698076	0.02	0.00	correct	correct
36	47.99006522	11.30695631	47.99006805	11.30695387	-	0.00	correct	correct
37	47.99007816	11.30694122	47.99008912	11.30693734	0.42	0.00	correct	correct
38	47.99009784	11.30691948	47.99011410	11.30691375	0.56	0.00	correct	correct
39	47.99012774	11.30688427	47.99013241	11.30687324	0.56	0.00	correct	correct
40	47.99015990	11.30684654	47.99018070	11.30683222	0.57	0.29	correct	correct
41	47.99019977	11.30681731	47.99023135	11.30680225	0.84	1.52	correct	correct

42	47.99025379	11.30678895	47.99026669	11.30679801	0.85	0.00	correct	correct
43	47.99027308	11.30677967	47.99028850	11.30677290	0.81	0.25	correct	correct
44	47.99029728	11.30676902	47.99031586	11.30676587	0.83	0.13	correct	correct
45	47.99034945	11.30674259	47.99037462	11.30673711	0.74	0.57	correct	correct
46	47.99040549	11.30671256	47.99043610	11.30670481	0.78	1.03	correct	correct
47	47.99046890	11.30668675	47.99049985	11.30667386	0.72	1.35	correct	correct
48	47.99052348	11.30666607	47.99054599	11.30666331	0.32	0.27	correct	correct
49	47.99057601	11.30664495	47.99060774	11.30663585	0.25	1.19	correct	correct
					AVERAGE: 0.411	AVERAGE: 0.26m	PRECISION: 93.9% RECALL: 100.0%	PRECISION: 91.8% RECALL: 100.0%

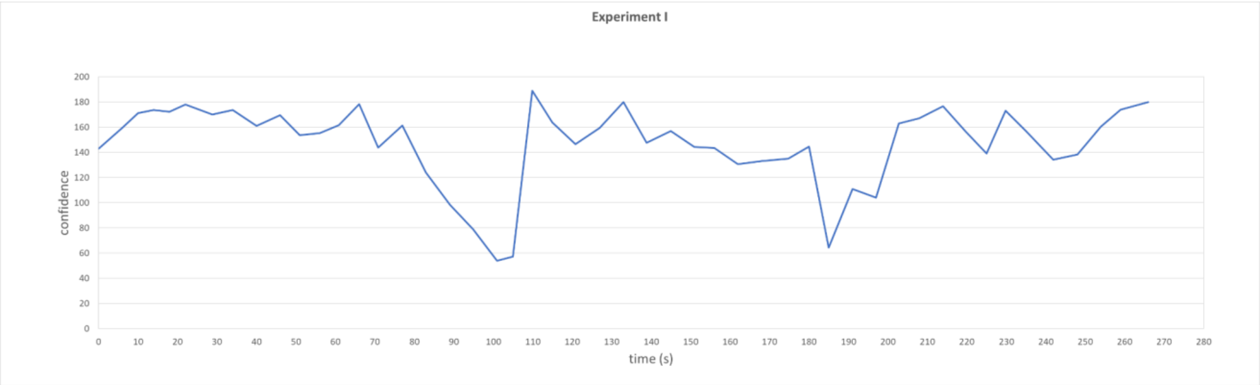

Figure S2. Confidence plot for Experiment I. The confidence values of our objective function over time, with an average confidence of c=147.1 and a confidence threshold of T=2.

Table S2 summarizes the quantitative measures and results, while Table S3 presents the sequence of detection results comparing our approach with the legacy method for Experiment II, conducted on 2024-05-16 at 5:20 a.m.

Table S2. Quantitative measures and results of Experiment II. GPS coordinates of ground-truth target positions and the swarm's estimates after detection. The ground distances between the swarm's target estimate and the target's ground-truth bounding box (in the case of the lying person: 1.25 m x1.8 m; in the case of the standing person: 0.8 m x 0.8 m). Note that a distance of 0 m indicates an estimate inside the target's bounding box.

detections	ground-truth target coordinates (center)		estimated target position coordinates		distance between ground-truth target (bounding box) and estimated target position (m)
	latitude	longitude	latitude	longitude	
1 (lying)	48.33662546	14.32141507	48.33663082	14.32142784	0.33
2 (standing)	48.33677860	14.3217020	48.33677685	14.32169552	0.08
					AVERAGE**:** 0.21m

Table S3. Detection results of Experiment II (our approach vs. legacy method). The sequence of detection results comparing our approach with the legacy method. Note, 'no' in the detection column indicates a false negative, while 'wrong' indicates a false positive. Note also, that in contrast to tracking moving targets (see Experiment I and III) detecting stationary targets requires only one correct detection after deployment. This is the case for our new approach in this experiment while the legacy method fails.

detections	detection (our approach)	detection (legacy method)
1 (lying)	no	wrong
	no	wrong
	no	wrong
	correct	wrong
2 (standing)	no	wrong
	correct	wrong
	correct	wrong
	correct	wrong
	correct	wrong

Table S4 and Figure S3 summarize quantitative measures and results of Experiment III, which was carried out on 2024-05-08 at 5:50 a.m.

Table S4. Quantitative measures and results of Experiment III. GPS coordinates of ground-truth target positions and the swarm's estimates for each PSO iteration. The ground distances between swarm's target estimates and the target's ground-truth bounding box (in the case of the persons: 1.25 m x 1.25 m). Note that a distance of 0 m indicates an estimate inside the target's bounding box. The detections with precision and recall of our approach and of the legacy method. Note also that 'no' in the detection column indicates a false negative, while 'wrong' indicates a false positive. False negatives are absent for the legacy method because the absolute threshold was fine-tuned to achieve the best possible results (i.e., an optimal threshold configuration that yields 100% recall). Note, that such fine-tuning is unrealistic under real-world conditions as anomaly cluster are unknown.

iteration	ground-truth target coordinates (center)		estimated target position coordinates		distance between ground-truth target (bounding box) and estimated target position (m)	detection (our approach)	detection (legacy method)
	latitude	longitude	latitude	longitude			
1	48.33670624	14.32194231	48.33671210	14.32195002	0.02	correct	correct
2	48.33670997	14.32191178	48.33670955	14.32191659	0.00	correct	correct
3	48.33671630	14.32185355	48.33671620	14.32186494	0.22	correct	correct
4	48.33672728	14.32181234	48.33672888	14.32182372	0.22	correct	correct
5	48.33673294	14.32179236	48.33673432	14.32180970	0.66	correct	correct
6	48.33674390	14.32174757	48.33676886	14.32222974	-	wrong	wrong
7	48.33676252	14.32170652	48.33675631	14.32172136	0.47	correct	correct
8	48.33677039	14.32167723	48.33676647	14.32168580	0.01	correct	correct
9	48.33677450	14.32167464	48.33677110	14.32167974	0.00	correct	correct

10	48.33677410	14.32167472	48.33677411	14.32167987	0.00	correct	correct
11	48.33677153	14.32166296	48.33677435	14.32167462	0.24	correct	correct
12	48.33676790	14.32161284	48.33677223	14.32162586	0.34	correct	correct
13	48.33675438	14.32156357	48.33676076	14.32157822	0.47	correct	correct
14	48.33673837	14.32151196	48.33674377	14.32152166	0.10	correct	correct
15	48.33671914	14.32144133	48.33672155	14.32147342	1.76	correct	correct
16	48.33669936	14.32140883	48.33669926	14.32144408	1.99	correct	correct
17	48.33669004	14.32139509	-	-	-	no	wrong
18	48.33668749	14.32138587	48.33668497	14.32171758	-	wrong	wrong
19	48.33668650	14.32139126	48.33669393	14.32141105	0.87	correct	correct
20	48.33668625	14.32139195	48.33668885	14.32140812	0.58	correct	correct
21	48.33668488	14.32138703	48.33668789	14.32140249	0.52	correct	correct
22	48.33667404	14.32137601	48.33668158	14.32139086	0.52	correct	correct
23	48.33666051	14.32133071	48.33666357	14.32134128	0.16	correct	correct
24	48.33664574	14.32129140	48.33665017	14.32130462	0.36	correct	correct
25	48.33664187	14.32127540	-	-	-	no	correct
26	48.33663080	14.32128668	48.33663287	14.32128309	0.00	correct	correct
27	48.33660421	14.32129477	48.33661128	14.32128928	0.16	correct	correct

28	48.33659252	14.32132756	48.33659242	14.32131626	0.21	correct	correct
29	48.33659350	14.32135717	48.33659380	14.32134568	0.23	correct	correct
30	48.33659688	14.32140559	48.33659696	14.32138880	0.62	correct	correct
31	48.33660515	14.32145768	48.33660026	14.32144903	0.02	correct	correct
32	48.33661089	14.32147946	48.33660610	14.32145921	0.88	correct	correct
33	48.33662374	14.32152300	48.33662414	14.32150521	0.69	correct	correct
34	48.33663634	14.32156115	48.33663524	14.32153798	1.09	correct	correct
35	48.33664862	14.32160528	48.33677152	14.32142225	-	wrong	wrong
36	48.33666279	14.32165967	48.33666166	14.32163599	1.13	correct	correct
37	48.33669343	14.32170521	48.33669035	14.32167642	1.51	correct	correct
38	48.33674337	14.32173326	48.33673705	14.32172075	0.32	correct	correct
39	48.33678032	14.32174679	48.33677232	14.32171743	1.58	correct	wrong
40	48.33681574	14.32178866	48.33680566	14.32175585	1.88	correct	correct
41	48.33685464	14.32179350	48.33684659	14.32177981	0.48	correct	correct
42	48.33689611	14.32178032	-	-	-	no	wrong
43	48.33693291	14.32176609	48.33691983	14.32174308	1.36	correct	correct
44	48.33696630	14.32169739	48.33695902	14.32168949	0.18	correct	correct
45	48.33698815	14.32163405	48.33697581	14.32163004	0.74	correct	correct

46	48.33702066	14.32160057	48.33708180	14.32158545	-	wrong	correct
47	48.33704207	14.32156839	48.33703503	14.32156270	0.15	correct	correct
48	48.33707180	14.32153639	48.33705220	14.32154742	1.57	correct	correct
49	48.33709149	14.32154730	-	-	-	no	correct
50	48.33711450	14.32155554	48.33711082	14.32155003	0.00	correct	correct
51	48.33713205	14.32155131	48.33712018	14.32154756	0.69	correct	correct
52	48.33713470	14.32155654	48.33713296	14.32154845	0.00	correct	correct
53	48.33712102	14.32162882	48.33711935	14.32162519	0.00	correct	correct
54	48.33710844	14.32169872	48.33710477	14.32169300	0.00	correct	correct
55	48.33708276	14.32174404	48.33708302	14.32173506	0.04	correct	correct
56	48.33702373	14.32181582	48.33702575	14.32180425	0.23	correct	correct
					AVERAGE: 0.53m	PRECISION: 92.3% RECALL: 92.3%	PRECISION: 89.3% RECALL: 100%

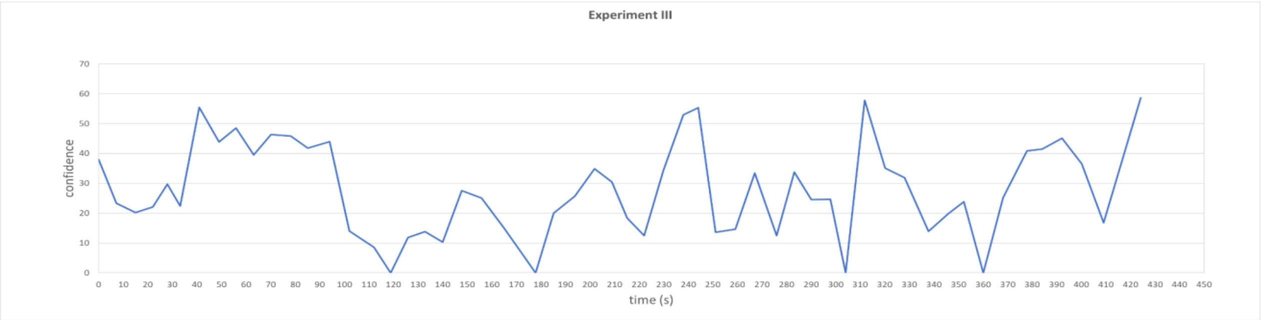

Figure S3. Confidence plot for Experiment III. The confidence values of our objective function over time, with an average confidence of *c*=27.6 and a confidence threshold of *T*=2. Note that the target was lost in 4 cases (*c*<*T*), but immediately re-detected in the next iteration.

Supplementary Note 4: Soft- and Hardware Architecture

We used DJI Mavic 3T (1xThermal, 1xRGB and 1xRGB Zoom camera) drones as platforms for our swarm. Also, we implemented a custom application running on the ARM64 Architecture-based DJI Pro / Plus Remote Controller (RC). The SoC (System on Chip package) on the RC is a Snapdragon 865 with 1 big, 3 med and 4 little cores, and the operating system is Android 10 (64-bit). Our custom application was implemented with DJI SDK v5.3 and has a communication interface for data transmission (telemetry, image/video data, and waypoint information) over a network to a custom Windows server application. Note that, although it also has image processing capabilities to execute anomaly detection, image registration, and image integration directly on the RC, these features were not used in our experiments. The server application receives video/image as well as telemetry data, and simultaneously sends waypoint data to up to ten drones. It also provides a Python interface for other applications to read and write this data. RCs are connected via a switch with the server PC, and data transmission is realized over ethernet. We use the Real-Time Streaming Protocol (RTSP) to stream the encoded YUV420SP (NV12) video data and embed telemetry data into RTSP's AAC audio packet. For waypoint information, we use Message Queuing Telemetry Transport (MQTT). The RCs and the drone communicate via Wifi, using DJI's proprietary OcuSync protocol.

On the Drone Side

For transporting the telemetry data simultaneously and synchronously with the video data, an AAC packet header is used to identify the telemetry data as an ACC audio packet (7 bytes ADTS AAC header + 250 bytes telemetry data) over RTSP. Telemetry data in this packet is in plain ASCII text format and is not encoded. RTSP video packets do not contain complete frames, but slices that contain only small image fragments and key frames (fill images) that are sent only periodically, depending on the configuration of the video encoder that generates the stream. A common key-frame interval for non-encapsulated HEVC video streams is 5 seconds. Due to the high video bit rate in our case, however, about 10 seconds are required. The key frames are needed by the video decoder to identify properly all slices that follow a key frame. Furthermore, codec-specific data (CSD-0 used in HEVC) is needed to identify the video parameters (e.g., width, height, color, video parameter set (VPS), sequence parameter set (SPS), and picture parameter set (PPS) information) by the decoder. They are sent once before the first key frame. Given that the video data is streamed at 30 Hz (fps) and the average slice package number to build an entire frame is approx. 6, the telemetry data is sent with ~60 Hz, irrespective of whether the drones' sensor hardware has the same sampling rate. For displaying the live video on the RC, the encoded video data was decoded with Android's MediaCodec HW decoder API. The parser of the optimized FFmpeg 6 library was integrated to rearrange incoming packages and feed them to the HW decoder. This enables fast RTSP streaming while the live video stream is shown on RC. For communication back to the drone, an open-source MQTT Server (Moquette Project) was implemented to receive commands such as target waypoints for autonomous flight.

On the Server Side

On the Windows server, a Microsoft Foundation Class (MFC) application was written in C++ using Visual Studio 2022. and linked statically with the FFmpeg 6 library. The libraries were built with Nvidia CUVID HW decoder support and include support for RTSP client connections. When connecting to a drone's RC, a new thread with real-time priority is spawned to handle the decoding process. A message system handles communication to and from the thread. Video streams from all drones are decoded simultaneously from the time

they are connected. When receiving the RTSP stream from a drone's RC, FFmpeg analyzes the stream and decides whether what is received is an audio or a video packet. For audio, packets no decoding is needed, as they are transmitted in plain ASCII text format and hold the telemetry data. Video packets, however, require decoding. All video package slices are assembled to full video frames, and GPU memory is reserved to send them to the internal video processing unit (VPU) of the graphics card. The output buffer returns the decoded frame in YUV420SP (NV12) format so that the resulting frame can be directly processed. On average, we achieved a round-trip time (including uploading waypoint data, downloading video and telemetry data, and video decoding) of approx. 80 ms per drone (for up to 10 simultaneously operated drones in the swarm) on the 24GB Nvidia Geforce RTX 4090 OC GPU used in our experiments (with 5th generation VPU). A Python wrapper interface (dynamic link library) was developed in C++ to exchange data (receiving video/telemetry data and sending waypoint data) between Python applications and the server. Each drone has its own shared memory area inside the Python wrapper library.

On the Client Side

A web-based map visualization client (implemented in JavaScript and HTML) can be connected through MQTT to the server for real-time mapping of the swarm and visualization of each drone's parameters (position, heading, full telemetry, and live video data). A digital zoom extends the limited zoom capabilities of conventional map services. The swarm control client (implemented in Python 3.7.9) communicates with the server through a python wrapper. Here, anomaly detection, image integration, particle swarm optimization, and our objective functions are executed, as explained in the main text of the paper. Its frontend allows hyperparameters to be adjusted, and displays in real time during flight the resulting (raw/RGB/thermal) integral images, RX integral images, the time-development of the objective function, and blob detections.

Hardware used in Field Experiments

In our field experiments, we used an autarkic and mobile ground station that can support up to 10 drone platforms in real time (downstreaming of video and telemetry data, and upstreaming of waypoint and control data). It consists of a high-end PC (5.8GHz Intel i9-13900KF processor (24 cores), 24GB Nvidia Geforce RTX 4090 OC GPU, 64GB RAM), a 16x Gigabit switch for fast internal data transmission between remote controllers, PC, and an external 5G link for networked RTK data transmission. A Bosch Power 1500 Prof battery unit provided power in the field for approximately 10 hours. A custom-built handheld networked RTK model (using an Arduino simpleRTK2B v1, a lightweight helical antenna for multiband GNSS, an XBEE bluetooth module, an Android phone running NTRIP Client and Geo Tracker, all built into a 3D-printed frame) was used for ground-truth target tracking. For RTK, the Austrian and German APOS services were used for experiments in Austria and Germany, respectively.

Supplementary Note 5: Downwash Tests

A series of downwash tests (cf. Figure S4) with our DJI Mavic 3T drones used in the swarm were carried out to investigate turbulence in overflight situations (as this may occur due to our vertical-separation collision-avoidance strategy). In our tests, one drone hovered at a constant distance of 5 m above ground, while a second drone maneuvered above it at various heights (Δh=1 m, 1.5 m, 2 m) and speeds (s=1,2,3,...10 m/s). We estimated height variations of the hovering drone from video recordings (see *Supplementary Movie 6 - Methods*). As expected, we observed that the higher s and Δh, the lower the turbulences due to the

downwash effect of the drone hovering above. The maximum downwash we observed (+/- 0.2 m) was for low speeds (below s=5 m/s) and short distances (Δh<2m). At no point in our trials did we observe higher turbulence. In fact, above s=5 m/s and above Δh= 2m, no significant turbulence was observed. Therefore, we add a 0.2 m safety margin to Δh in our altitude separation for cases in which our drones fly at less than 5 m/s and closer together than 2 m.

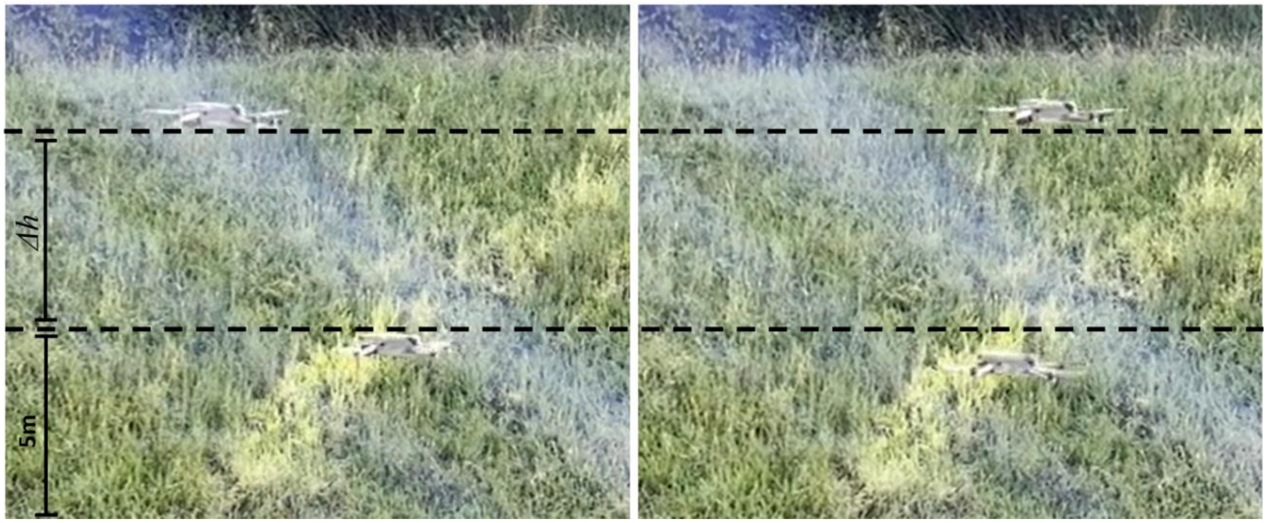

Figure S4. Downwash Test Setup. A drone hovering at 5 m above ground (bottom) is overflown by another drone (top) at various distances Δh and speeds s.

SUPPLEMENTARY REFERENCES

1. Isaksen, A., McMilan, L. & Gortler, S. J. Dynamically reparameterized light fields. In *Proc. 27th Annu. Conf. Comput. Graph. Interact. Tech.* 297–306 (ACM, New York, USA, 2000).
2. Levoy, M. & Hanrahan, P. Light field rendering. In *Proc. 23rd Annu. Conf. Comput. Graph. Interact. Tech.* 441–452 (ACM, New Orleans, USA, 1996).
3. Kurmi, I., Schedl, D. C. & Bimber, O. Airborne optical sectioning. *J. Imaging* **4**, 102 (2018).
4. Reed, I. S. & Yu, X. Adaptive multiple-band CFAR detection of an optical pattern with unknown spectral distribution. *IEEE Trans. Acoust.* **38**, 1760–1770 (1990).
5. Chang, C. I. & Chiang, S. S. Anomaly detection and classification for hyperspectral imagery. *IEEE Trans. Geosci. Remote Sens.* **40**, 1314–1325 (2020).
6. Shyu, M. L., Chen, S. C., Sarinnapakorn, K. & Chang, L. A novel anomaly detection scheme based on principal component classifier. In *Proc. IEEE Foundations New Directions Data Mining Workshop* 172–179 (IEEE, Melbourne, USA, 2003).
7. Bishop, C. M. & Nasrabadi, N. M. Pattern recognition and machine learning. *J. Electron. Imaging* **2**, 1122–1128 (2006).
8. Carlotto, M. A cluster-based approach for detecting man-made objects and changes in imagery. *IEEE Trans. Geosci. Remote Sens.* **43**, 374–387 (2005).
9. Breunig, M., Kriegel, H., Ng, R. & Sander, J. LOF: identifying density-based local outliers. In *Proc. 2000 ACM SIGMOD Int. Conf. Manage. Data* 93–104 (ACM, Dallas, USA, 2000).
10. Seits, F., Kurmi, I. & Bimber, O. Evaluation of color anomaly detection in multispectral images for synthetic aperture sensing. *Eng.* **3**, 541–553 (2022).
11. Cheng, T. *et al.* Yolo-world: real-time open-vocabulary object detection. In *Proc. IEEE/CVF Conf. Comput. Vis. Pattern Recognit.* 16901–16911 (2024).
