# Supplementary material for: An autonomous drone swarm for detecting and tracking anomalies among dense vegetation
Source: Commun Eng. 2025 Nov 27;4:205. doi: 10.1038/s44172-025-00546-8 (PMC12660990; doi:10.1038/s44172-025-00546-8)
Supplement: Supplementary file 3 — Description of Additional Supplementary Files [file 44172_2025_546_MOESM3_ESM.pdf]

Description of Additional Supplementary Files

File name- Supplemental Movie 1

File description – Introduction and Previous Work (Simulation).
File name- Supplemental Movie 2

File description - Detection, Tracking, and Classification of Moving Targets in Sparse Forest (Experiment I).
File name- Supplemental Movie 3

File description - Detection of Localized Targets in Dense Forest and Swarm Deployment (Experiment II).
File name- Supplemental Movie 4

File description - Detection and Tracking of Moving Targets in Dense Forest (Experiment III).
File name- Supplemental Movie 5

File description - Summary and Concluding Remarks.
File name- Supplemental Movie 6

File description - Downwash Tests, Hard- and Software Framework, Take-off and Landing Procedures.
